# Editorial: Advancements in feline oncology: diagnosis, treatment, and management of domestic cat cancers

**DOI:** 10.3389/fvets.2025.1694132

**Published:** 2025-10-01

**Authors:** Rodrigo dos Santos Horta, Antonio Giuliano, Fernanda Vieira Amorim da Costa

**Affiliations:** ^1^Department of Veterinary Medicine and Surgery, Veterinary School, Universidade Federal de Minas Gerais, Belo Horizonte, Brazil; ^2^Harvest Veterinary Oncology Center, Kowloon, Hong Kong SAR, China

**Keywords:** neoplasm, feline, epidemiology, electrochemotherapy, prognosis, histiocytic disorders, Newcastle Disease Virus (NDV)

The domestic cat is one of the most beloved and widely spread companion animals with a population of approximately 1 billion individuals, although more than half of those comprise stray cats. Their popularity as pets has grown globally, largely because they are considered low-maintenance, quiet, and affordable. Currently, the largest populations of domestic cats are found in the United States, China, Russia, Germany, France, Brazil, the United Kingdom and Italy. Cats are more popular than dogs in Russia and Japan, reflecting cultural preferences and lifestyle factors that favor feline companionship.

The life expectancy of cats varies significantly depending on several factors, including age, sex, neuter status, breed and geographical location. In the United Kingdom, the average life expectancy is estimated at 11.7 years, with road traffic accidents representing the leading cause of death, followed by renal disease, cardiovascular disease and neoplasia (1).

Neoplasia is one of the main causes of mortality in adult and senior domestic cats and it represents an increasing health concern. Moreover, it represents a major health risk to young cats living in FeLV-endemic areas, as it dramatically increases the likelihood of developing aggressive forms of lymphoma and leukemia (2, 3).

In this context, feline oncology has emerged as an important field in veterinary medicine due to the growing global cat population and rising prevalence of neoplastic diseases. Unlike other companion animals, cancer in cats exhibit unique biological and clinical behaviors that demand species-specific diagnostic and therapeutic strategies. This Research Topic brings together cutting-edge studies that address critical challenges in the diagnosis, treatment, and understanding of feline cancers. The contributions presented here encompass epidemiological analyses, novel prognostic markers, and innovative therapeutic approaches, collectively advancing our ability to provide more effective and tailored oncological care for feline patients.

Giugliano et al. provided an extensive epidemiological analysis of *mammary gland, skin, and soft tissue tumors in cats* using 20 years of data from Italy's Liguria region. The researchers analyzed a database of 4,399 registered tumors. Their population-based study confirmed the predominance of mammary gland tumors in intact females and a higher frequency of skin and soft tissue tumors in neutered cats, regardless of sex. Notably, the study found a correlation between environmental cadmium pollution and cancer incidence, underscoring the role of cats as sentinels of environmental health risks. This research bridges the fields of feline oncology and public health, supporting the One Health perspective and comparative oncology models.

Diagnosis is the first step in planning a cancer therapy or strategies to reduce its incidence. However, prognosis is also fundamental to decision-making processes regarding treatment. dos Santos et al., investigated perineural invasion (PNI) in feline squamous cell carcinoma (SCC), treated with electrochemotherapy (ECT). A total of 24 cats were included in the study: 21 with skin SCC and three with oral lesions. PNI was identified in 8/24 (33%) cats and it was significantly associated with local recurrence following ECT, with all PNI-positive cats experiencing relapse, compared to 5/16 (31.2%) of cats without PNI invasion (*p* = 0.03). This study introduces PNI as a possible prognostic biomarker and calls for further prospective investigations into PNI, which may eventually support refinements in treatment planning, surveillance, and client communication.

The potential of ECT as a local control strategy was further substantiated in the original research by de Sena et al.. In the realm of rare and treatment-resistant neoplasms, this Research Topic presents a compelling case report on feline progressive histiocytosis (FPH), a poorly understood and infrequently diagnosed condition originating from interstitial dendritic cells. A 6-year-old spayed female mixed-breed domestic cat, was presented with disseminated lesions that were diagnosed as FPH. A multimodal therapeutic approach combining electrochemotherapy, toceranib phosphate, and chlorambucil led to complete remission and long-term survival, despite the cat's previous unresponsiveness to the maximum tolerated doses of lomustine and doxorubicin. This case not only highlights the clinical utility of electrochemotherapy in cutaneous neoplasms but also opens new discussions about individualized protocols for rare cancers.

In this context, Alves et al. investigated the oncolytic potential of Newcastle Disease Virus (NDV) as a novel treatment for feline lymphoma, a common and often challenging cancer in cats that is often related to FeLV, especially in young cats living in endemic regions. The researchers utilized a modified NDV strain, NDV-GFP, to infect and induce cell death in feline thymic lymphoma cells infected with FeLV and cultured in a laboratory setting. The results demonstrated that NDV-GFP successfully replicated within these cancer cells and reduced their viability in a dose-dependent manner, suggesting that it could serve as an effective virotherapy. This preliminary *in vitro* evidence highlights NDV as a promising candidate for developing new therapeutic strategies for feline lymphoma, especially given the limitations and side effects associated with conventional chemotherapy. This investigation underscores the importance of exploring non-traditional modalities that could overcome the limitations of conventional therapies.

Taken together, these contributions advance our understanding of feline oncology by bridging basic research and clinical applications. They highlight the diversity and complexity of the challenges faced in feline cancer care—including large-scale epidemiological trends, identification of recurrence predictors, multimodal strategies for managing rare tumors, and development of innovative virotherapy approaches.

We anticipate that these studies will stimulate further research into species-specific treatment protocols, the development of predictive biomarkers, and improved diagnostic tools tailored to feline patients. Ultimately, as the field of veterinary oncology continues to evolve, so does our capacity to enhance not only the lifespan but also the quality of life of cats affected by cancer, fostering a more personalized and evidence-based approach to feline cancer management.

## References

[1] Teng KTY Brodbelt DC Church DB O'Neill DG. Life tables of annual life expectancy and risk factors for mortality in cats in the UK. J Feline Med Surg. (2024) 26:1098612X241234556. 10.1177/1098612X241234556 38714312 PMC11156239

[2] Kent MS Karchemskiy S Culp WTN Lejeune AT Pesavento PA Toedebusch C. Longevity and mortality in cats: a single institution necropsy study of 3108 cases (1989-2019). PLoS ONE. (2022) 17:e0278199. 10.1371/journal.pone.0278199 36580443 PMC9799304

[3] Biezus G Grima de Cristo T Flores Koehler CM de Quadros Kaveski FY Sarria Viana Miranda T Ferian PE. Survival analysis and clinical abnormalities in cats with progressive or regressive feline leukemia virus (FeLV) infection in Brazil. PLoS ONE. (2025) 20:e0322691. 10.1371/journal.pone.0322691 40591622 PMC12212530

